# An Intelligent Optimization Algorithm for Constructing a DNA Storage Code: NOL-HHO

**DOI:** 10.3390/ijms21062191

**Published:** 2020-03-22

**Authors:** Qiang Yin, Ben Cao, Xue Li, Bin Wang, Qiang Zhang, Xiaopeng Wei

**Affiliations:** The Key Laboratory of Advanced Design and Intelligent Computing, Ministry of Education, School of Software Engineering, Dalian University, Dalian 116622, China; qiangy772@gmail.com (Q.Y.); bencaocs@gmail.com (B.C.); daisywowa@gmail.com (X.L.); xpwei@dlu.edu.cn (X.W.)

**Keywords:** DNA storage, DNA coding design, HHO algorithm, OBL

## Abstract

The high density, large capacity, and long-term stability of DNA molecules make them an emerging storage medium that is especially suitable for the long-term storage of large datasets. The DNA sequences used in storage need to consider relevant constraints to avoid nonspecific hybridization reactions, such as the No-runlength constraint, GC-content, and the Hamming distance. In this work, a new nonlinear control parameter strategy and a random opposition-based learning strategy were used to improve the Harris hawks optimization algorithm (for the improved algorithm NOL-HHO) in order to prevent it from falling into local optima. Experimental testing was performed on 23 widely used benchmark functions, and the proposed algorithm was used to obtain better coding lower bounds for DNA storage. The results show that our algorithm can better maintain a smooth transition between exploration and exploitation and has stronger global exploration capabilities as compared with other algorithms. At the same time, the improvement of the lower bound directly affects the storage capacity and code rate, which promotes the further development of DNA storage technology.

## 1. Introduction

From paper recording to the current U disk, as well as digital storage such as hard disk to more sophisticated quantum media, storage is an indispensable way to preserve history and knowledge for later use. Due to the limitation of the storage density of electromagnetic media, the development of digital storage technology has been slow, and technology based at the molecular level is rapidly developing synchronously. With its large storage capacity, high density, and long-lasting storage capacity, DNA data storage has become one of the most advanced technologies for long-term data storage [1]. A DNA molecule consists of four basic nucleotides, including “A”, “T”, “C”, and “G” [2], which form a double helix through complementary base pairing (also called hybridization) of bases. In terms of structure, A, and T are bonded by a double hydrogen bond, and C is bonded to G by three hydrogen bonds. Moreover, the specific double helix structure and the mutual stacking of these bases make the storage capacity more than 1000 times larger than that of silicon-circuit-based devices [3]. This high storage density allows up to 455 EB bytes of data per gram of single-stranded DNA [4] and the binary storage capacity of DNA molecules is about 4.2 × 1021 bits per gram, which is 420 billion times that of conventional storage media [5]. Attracted by the above characteristics, the feasibility of DNA storage has been confirmed in several experiments [4,5,6,7], and DNA storage technology is developing rapidly and is expected to become the most prominent storage method in the near future.

The main technologies used in DNA storage include DNA synthesis techniques [8] for “coding” and DNA sequencing techniques [9] for “decoding”. Synthesis refers to the process of converting digital information into “A”, “T’, “C” or “G” sequences using a pre-developed coding scheme, and then synthesizing these sequences into oligonucleotides or long DNA fragments. The process of sequencing is the opposite of this. However, the quality of the coding sequence in the synthesis process directly affects the efficiency of these reactions and the success of decoding, so it is necessary to construct a robust coding sequence. In response to this, our predecessors have done a lot of work on DNA storage. Baum [10] first proposed to construct a DNA storage model, which could be used to construct DNA memory with a large storage capacity, thus laying the foundation for DNA storage technology research. This subfield has continued to grow slowly over the past few years. Clelland et al. [11] further developed a DNA-based double steganographic technique for encoding secret messages in DNA. An additional paper [12] gave three important reasons for why DNA molecules are an ideal media for long-term information storage. Other authors [13] described a memory model made of DNA, called Nested Primer Molecular Memory (NPMM), and proved its feasibility through experiments. Ailenberg et al. [14] described an improved Huffman coding method for information storage using DNA, and their experiments showed that this method was suitable for automatic information retrieval. Additionally, Church et al. [4] wrote 5.27 Mb bits of files into DNA microchips and successfully used DNA sequencing to decode these files, which was an important milestone in storage history. After this, storage research using DNA has entered an era of rapid development. Goldman et al. [6] used a three-element Huffman-encoded synthetic DNA sequence on 739K different format files and achieved a sequencing accuracy of 100%. Yazdi et al. [15] described the first DNA storage architecture that allowed random access to blocks of data and the rewriting of information. This innovation ensured the reliability of the data while increasing the data storage capacity. Later, Bornholt et al. proposed an archival storage system-based architecture and a new coding scheme, which provided random access and controllable redundancy for DNA storage [16]. Blawat et al. [17] developed an efficient and robust forward error correction scheme for DNA channels, and this solution could deal with insertions, deletions, and swap errors.

A lot of work-related to DNA storage has appeared in the literature over the past three years, which shows its practical value. For example, Erlich et al. proposed a DNA fountain code storage strategy, successfully storing 2.4 × 10^6^ bits of bytes in a DNA oligonucleotide sequence, which was perfectly decoded [7]. Yazdi et al. [18] demonstrated, for the first time, a method for implementing a portable random-access platform using a nanopore sequencer in practice, which reduced errors in sequencing and enabled random access of any part of the encoded data. Soon after, Gabrys et al. [19] proposed a new family of codes called asymmetric Lee distance (ALD) codes to correct errors and give an upper bound on the size of the code under this metric. In order to avoid the appearance of long homopolymers in DNA storage, Immink et al. proposed a sequence replacement method for k-constrained q-ary data, which produced a significant improvement in coding redundancy [20]. Organick et al. [21] used more than 13 million DNA oligonucleotides to encode and store 35 different files (more than 200 MB of data) and used a random-access method to recover each file individually without errors. Later, Yazdi et al. [22] introducee the notion of weakly mutually uncorrelated (WMU) sequences and presented a variety of constructs for WMU codes in real-world storage applications. Song et al. [23] proposed a coding method for converting a binary sequence into a DNA basic sequence that satisfied the run-length constraint and the GC-content constraint properties, and this system achieved a rate of 1.9 bits per DNA base with low encoding/decoding complexity and limited error propagation. Carmean et al. [24] developed new ways of storing DNA using electronic biomixing systems. In the same year, Heckel et al. [25] used experimental data to quantitatively and qualitatively understand DNA storage channels to help guide the design of future DNA data storage systems. Subsequently, Limbachiya et al. [26] used altruistic algorithms based on three constraints to construct a DNA code set in storage that significantly improved the lower-bound results. Wang et al. [1] proposed a new content-balanced run-length limited code that satisfied both the maximum homopolymer operating limit and the equilibrium GC-content limit, ensuring local and global stability. Shortly after this, Takahashi et al. [27] developed an automated end-to-end DNA data storage device and demonstrated a 5 byte automatic write, store and read cycle that could be extended as new technologies emerge. Another paper [28] conducted a preliminary study of DNA storage in representative living organisms, such as E. coli, yeast, and Arabidopsis, and finally found that digital information can be stored and stably transmitted on DNA using these living organisms. Recently, Ceze et al. [29] summarized the mainstream technologies and challenges facing in the field of DNA storage, which is of great significance for future research. Wang et al. [30] monitored the long-term storage of DNA using ddPCR. Anavy et al. [31] encoded data using fewer synthesis cycles, and this grouped encoded 6.4 MB of data into composite DNA, reducing the number of synthesis cycles used per unit of data by 20%. Immediately afterward, Deng et al. [2] proposed a hybrid coding scheme consisting of an improved variable-length run-length limited (VL-RLL) code and an optimized prototype low-density parity-check (LDPC) code, and used an improved external information transmission algorithm (EXIT), which showed that the proposed hybrid coding scheme stored 1.98 bits per nucleotide (bits/nt), with only a 1% gap from the upper boundary (2 bits/nt).

The reason why DNA coding sequences as “information” in storage cannot be randomly selected is that DNA molecules can produce undesired false positives and false negatives during hybridization. Therefore, it is necessary to add correlation constraints (called constraint coding) to optimize the coding sequence to enhance the robustness of any sequence. Robust coding sequences are important for “reading” and “writing” in storage, but the number of these different length sequences directly limits the capacity and efficiency of storage. We have sought to increase the lower bound of the constraint coding sequence of different lengths as much as possible. Commonly used constraints are the No-runlength constraint, the GC-content constraint, and the Hamming distance constraint.

## 2. Constraints on DNA Codes

To store data in DNA more reliably, different constraints must be imposed on the sequences used in a DNA-coding set. These constraints can enhance the robustness of the sequence for better data storage. In order to design a DNA coding with No-runlength constraint and constant GC-content, constraint encoding is considered here, represented by q (*n*, *M*, *d*), where n represents the length of a sequence, M represents a symbol, and d represents the Hamming distance. Under these constraints, we tried to build as many constraint code sets as possible of different lengths as follows:

• No-runlength constraint

For q (*n*, *M*, *d*), a No-runlength constraint [26] means that two adjacent base elements at any position in the sequence are different. For example, in the base sequence *CTAACG*, the *A* base appears consecutively multiple times, violating this constraint, which can result in an increase in the bit error rate during decoding. The existence of a No-runlength constraint is beneficial to avoid the occurrence of homopolymers, so as to avoid the formation of secondary structure and prevent file corruption leading to decoding failure. For a DNA sequence containing n bases (*b*_0_, *b*_1_, *b*_2_… *b_n_*), this constraint is defined as follows by Equation (1):

(1) $$b_{i}\neq b_{i-1}i\in [1,n]$$

• GC-content constraint

The GC-content constraint [32] in a q (*n*, *M*, *d*) set typically requires that the total content of bases G or C be 50% of the code length n, as a constant GC-content maintains the stability of a DNA sequence.

For a sequence s, the GC-content is defined as GC(s), and here it is equal to 50%. We used the following formula by Equation (2) to calculate the GC-content:

(2) $$GC\left(s\right)=\frac{G+\left[C\right]}{\left[s\right]}$$

• Hamming distance constraint 

Assuming there is a pair of sequences *x* and *y* of the same length (*x*! = *y*) in the q (*n*, *M*, *d*) set, the sum of the number of different base elements at the same position is called the Hamming distance [33], and is expressed by H (*x*, *y*). Satisfying H (*x*, *y*) ≥ *d*, *d* is a defined threshold.

The Hamming distance is considered because it can discriminate between two sequences, and sequences with too much similarity lead to nonspecific hybridization reactions. At the same time, these can correct substitution errors [33] and play a crucial role in data resilience [26]. The larger the Hamming distance, the smaller the similarity between two sequences and the more stable the sequences are. The Hamming distance is calculated as follows by Equation (3):

(3) $$H(x,y)=\sum_{i=1}^{n}h(x_{i},y_{i}),h(x_{i},y_{i})=\{\begin{array}{c} 0,x_{i}=y_{i}\\ 1,x_{i}\neq y_{i} \end{array}$$

In this paper, we only considered a quaternary code, namely “A-0, G-1, C-2, T-3”. A quaternary code has better storage capacity and coding rate as compared with a ternary code. Then, the proposed NOL-HHO algorithm was used to find more sequences that satisfy the No-runlength constraint, the GC-content, and have a large Hamming distance.

## 3. Improve the Constrained DNA-Sequence Lower Bound’s Method

Obtaining an optimally constrained DNA-sequence lower bound is a complex combinatorial optimization problem and has always been a bottleneck in the DNA storage field. However, in recent years, the metaheuristic optimization algorithm has become very suitable for such problems, due to its fast convergence on an approximate optimal solution. In this paper, we use the Harris Hawk optimization algorithm proposed by Mirjalili et al. [34]. The Harris Hawk optimization algorithm has been successfully applied to many practical applications due to its complete attack strategy and has achieved good results [35,36,37], but there are still some disadvantages that make it subject to local problems. We used a nonlinear control parameter strategy to improve the linear convergence factor, to maintain a smooth transition of algorithm exploration and exploitation, increase population diversity, and to accelerate convergence. Then, we introduced a new random opposite-learning strategy in conjunction with the Harris Hawk optimization algorithm. This combination caused the population to jump out of local optima and enhanced the global exploration capability of our algorithm. We tested the improved algorithm using 23 widely used benchmark functions such as single-peak and multi-peak functions, and many of these reached global optimal values, demonstrating the effectiveness of our algorithm improvements. Subsequently, rank-sum tests and function tests of different dimensions were carried out. The experimental results showed that our algorithm had a stronger global grasp as compared with other classical algorithms and original algorithms. Finally, the proposed algorithm was applied to construct a lower bound of a constrained coding set for DNA storage and we compared this with a set constructed using the altruistic algorithm [26]. The results further show that even in practical applications, the constructed algorithm had strong superiority.

### 3.1. The Original Algorithm (HHO)

The Harris hawks optimization algorithm (HHO) is a population-based metaheuristic optimization algorithm that was proposed by Mirjalili et al. [34]. Its main inspiration came from the cooperative predation between the hawks in the Harris area of the United States and the different chasing strategies for prey, also referred to as the ‘surprise pounce’. The pounce refers to other hawks’ members from different directions that help the leader quickly converge on a given prey (i.e., surprise it and catch it without a response). If a leader gets lost in the middle of the predation, the other top members immediately replace the leader’s role. This slow-surrounding strategy makes the chased rabbit confused and tired. When Harris hawks find that a rabbit is exhausted, the leader immediately attacks and the rabbit is eventually captured by the hawk population. This complete strategy makes the HHO algorithm have better results than most other algorithms in dealing with theoretical tests and practical engineering optimization problems.

The HHO algorithm mainly includes two stages of exploration and exploitation. The exploration phase uses two different equal probability strategies to update the position of the hawks. The hawks randomly choose a tall habitat as their location so they can track prey. The hawks can also update locations based on the location of other family members and prey so that they can be closer to other members when they are assisting the team. The hawks in the exploitation phase use four equal probability attack strategies to capture prey, based around the prey energy change and escape mode. For example, if the soft besiege and hard besiege strategy fail, the hawks simulate the prey escape movement and quickly dive around the prey to adjust their flight direction and position for a new round up.

The escape energy of prey is an important factor in maintaining a smooth transition between exploration and exploitation. The conversion between different attack strategies during the development phase is also based on this, as expressed by ”*E*” in the formula as follows by Equations (4) and (5):

(4) $$E_{1}=2*\left(1-t/T\right)$$

(5) $$E_{1}=\left(2*r_{1}-1\right)*E_{1}$$

where *E* represents the escape energy of the prey, *r*_1_ is a random number between (0, 1), *T* is the maximum number of iterations, *t* is the current number of iterations, and *E*_1_ is the convergence factor that decreases linearly with the number of iterations, with an interval [2,0]. When | *E*| ≥ 1, HHO explores the search space to determine promising areas. Conversely, when |*E*| is smaller than 1, the strategy is used to improve the local search efficiency. Detailed mathematical modeling of all HHO strategies can be found in [34].

### 3.2. The Improved Algorithm (NOL-HHO)

Although the HHO algorithm has a sound exploration and exploitation strategy, the results of the 23 test functions in the original paper showed that it still had room for improvement, as only the *F*_9_, *F*_10_, *F*_11_, *F*_17_, *F*_19_, and *F*_23_ test functions reached global optimal values [34]. After analysis, we found that the linear oscillation of the escape energy was flawed. In the second half of the iteration (after 250 iterations), the absolute value of *E* was always less than 1. That is, although the current region may not be ideal, the search agent still exploited the region later in the iteration, so there was no guarantee that the group would gather near the global optimal value at the end of the exploration phase, leading to premature convergence on a local optimum. For this, we propose a nonlinear control parameter strategy and a random opposition-based learning strategy to improve the original algorithm, as detailed below.

#### 3.2.1. Nonlinear Control Parameter Strategy

**Figure 1:**
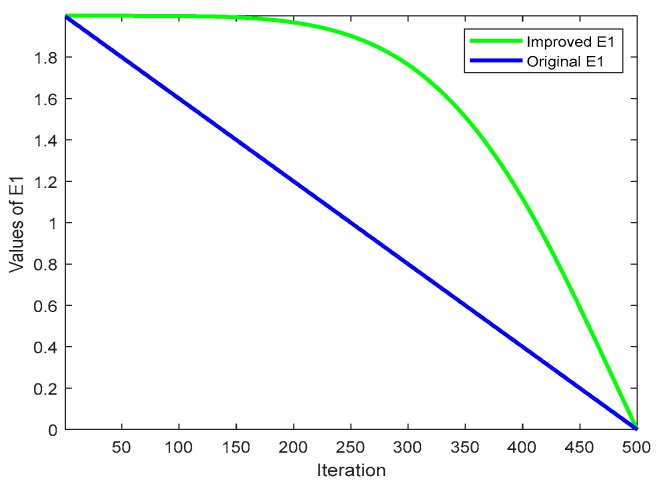
Change curve of control parameter **E_1_**.

**Figure 2:**
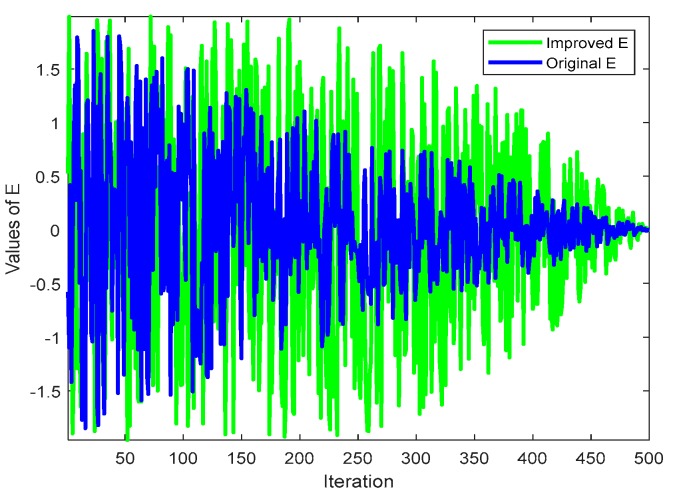
Dynamic curve of escape energy **E**.

The main factor determining the linear decline of the escape energy *E* is *E*_1_ in HHO, and the formula is as shown in Equation (4). In the first half of the iteration, a promising area is explored. In subsequent iterations, the area does not jump to the promising space even if it is not ideal, and only a detailed search is performed of this area, which can cause the algorithm to stall in a bad search space. Moreover, linear changes do not truly reflect changes in prey energy consumption and the actual optimization of the search process. The nonlinear control parameter strategy has been applied to the improvement of algorithms [38]. and the test results were better than those of linear strategies. This work uses a new nonlinear control parameter strategy for *E*_1_ to overcome the above shortcomings and improve the global performance. The refined *E*_1_ formula is as follows by Equation (6):

(6) $$E_{1}=\left(b_{fin}-b_{ini}\right)*\frac{1}{1-e}*\left[1-\frac{e}{1-e^{{\left(\frac{t}{T}\right)}^{5}}}\right]$$

where *b_fin_* and *b_ini_* represent the final value 2 and the initial value 0 of the control parameter, respectively, *t* represents the current number of iterations, and *T* represents the maximum number of iterations. We compared the results of *E*_1_ and *E* before and after the improvement, as shown in Figure 1 and Figure 2. It can be seen from Figure 1 that after *E*_1_ was utilized, the deceleration speed was slower in the previous iteration, which can increase the global search ability and avoid the algorithm falling into local optima. The later iterative decrement speed was fast, and the algorithm local search ability was increased, thereby improving the overall convergence speed. For *E* in Figure 2, the improvement of *E*_1_ enhanced the disturbance, but it was still very good at exploration in the later stage of iteration. If the search agent entered an undesired area, the population was not be confined to that area, but rather was designed to seek out more promising areas to avoid falling into premature local optima.

#### 3.2.2. Random Opposition-Based Learning Strategy

In 2005, Tizhoosh et al. [39] first proposed an opposition-based learning strategy. This was a new concept in computational intelligence. In recent years, it has been widely used to enhance various optimization algorithms [40,41,42], and the test results prove its effectiveness. The main idea is to calculate the inverse solution of a feasible solution in addition to finding a feasible solution in a group, thus, providing another opportunity to find a candidate solution closer to the global optimal value.

This strategy functions as follows:

**Theorem** **1.** *Contrary to what is described in number, let x ∈ [a, b] be a real number. The opposite of x is defined by the following [39]:*

(7) $$\hat{x}=a+b-x$$

And the definition of high dimensions is as follows:

**Theorem** **2.** Let X_j_ = (x_1_, x_2,_ …, x_D_) be a point in a D-dimensional space, where x_j_ ∈ [a_j_, b_j_] and j ∈ 1, 2,…, D. The opposite point X_j_* = (x_1_*, x_2_*,…, x_D_*) is defined by:

(8) $$x_{j}^{*}=a_{j}+b_{j}-x_{j}$$

Although this adds opportunity to the global optimal proximity detection, if both the feasible solution and the opposing solution deviate from the global optima, and the algorithm still falls into a local optimization. To enrich population diversity and increase opportunities, we propose a new random opposition-based learning strategy, defined as “ROL” as follows by Equation (9):

(9) $${\hat{x}}_{j}=a_{j}+b_{j}-rand\left(\right)\times x_{j},1,2,\ldots ,n$$

“ROL” can move the current candidate to a new search space to jump out of a local optimization, meaning we can calculate the fitness function values of individuals before and after a random opposition-based learning strategy, and use the best selection mechanism to select N better individuals.

We refer to the HHO algorithm that combines the above two strategies as the “NOL-HHO” algorithm. The former nonlinear control parameter had a good ability to balance global and local searches, while the latter “ROL” strategy guides the population to jump out of a local area to exploit more promising areas, both of which greatly improve the performance of HHO.

### 3.3. NOL-HHO Algorithm’s Pseudocode and Flow Char

**Figure 3:**
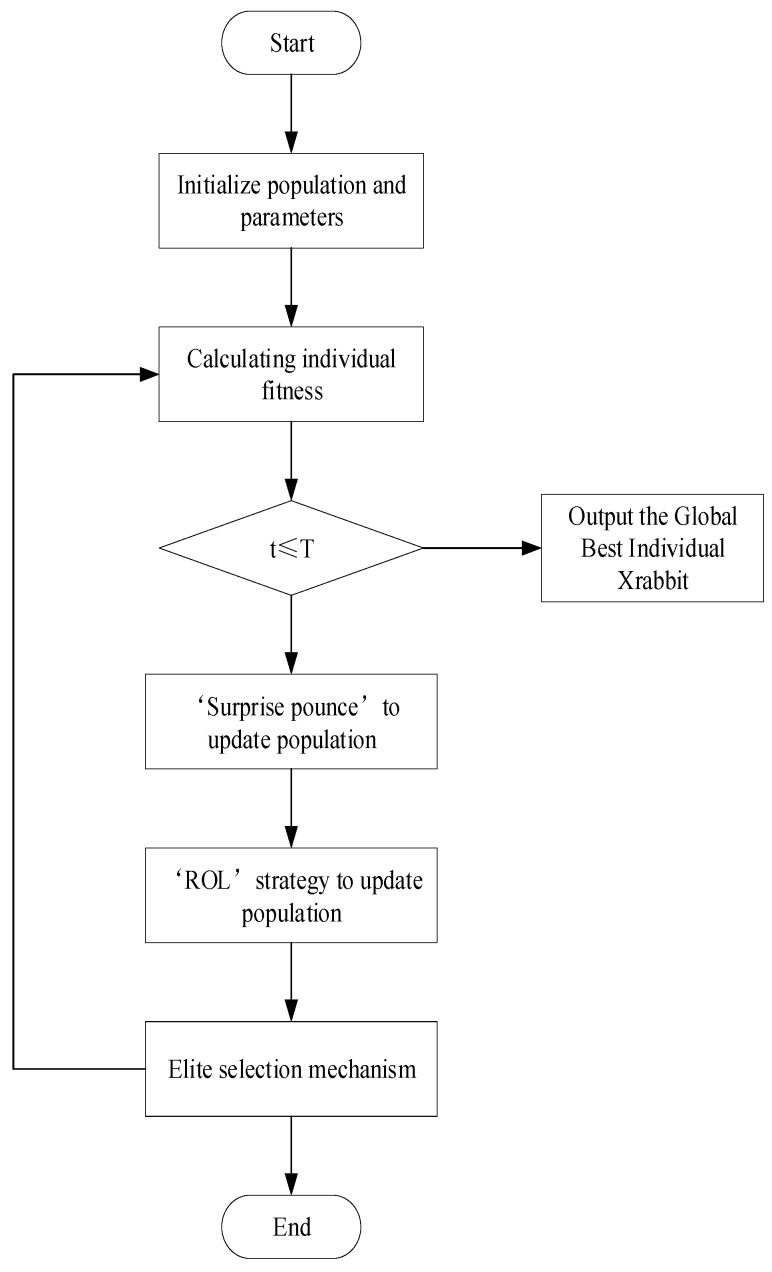
Flow chart of NOL-HHO algorithm.

**Figure 4:**
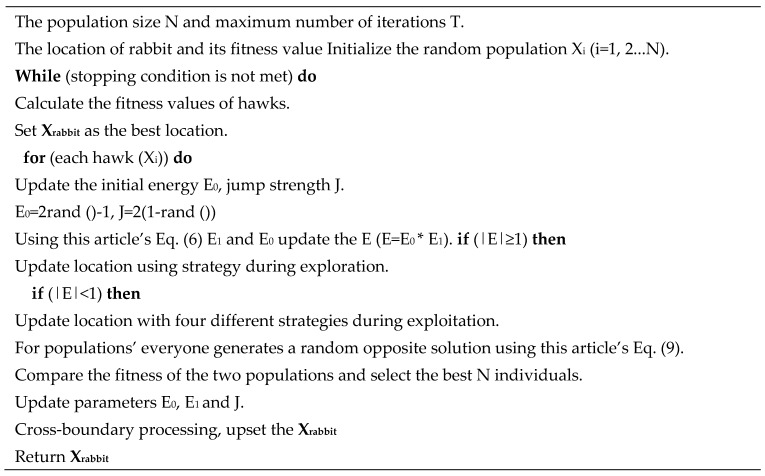
Pseudocode of NOL-HHO algorithm.

The following is a flowchart and detailed pseudocode for the NOL-HHO algorithm, as shown in Figure 3 and Figure 4. For the formula of the exploration and exploitation process in pseudocode, please refer to the original HHO paper [34].

## 4. Test of the Proposed Algorithm NOL-HHO

### 4.1. Benchmark Test Functions

To illustrate that the improved NOL-HHO algorithm was superior to the original algorithm and other algorithms in handling various mathematical benchmark tasks, we tested 23 benchmark functions that are widely used. Functions can be divided into three categories according to different function types [43,44], including unimodal benchmark functions (*F*_1_–*F*_7_), multimodal benchmark functions (*F*_8_–*F*_13_), and fix-dimension multimodal benchmark functions (*F*_14_–*F*_23_). The equations for the different types of functions are listed in the supplementary file., where Dim represents the dimension of the functions, range is the boundary of the search space of the corresponding functions, and *f_min_* represents the global optimal value.

Since unimodal functions have only one global optimal value and no local optimal values, they can be applied to the benchmark exploitation capability of the test algorithm. The specific unimodal benchmark functions are shown in Table S1. Compared with unimodal functions, multimodal benchmark functions have a large number of local optimal values and increase with an increase of the processing dimension, which enables the multimodal benchmark functions to be used to test the exploration ability of our algorithm and its ability to jump out of a local optimum. The multimodal benchmark functions considered are shown in Table S2. The fix-dimension multimodal benchmark functions are the same as the multimodal benchmark functions, there is only one global optimal value, and many locally optimal solutions. However, the difference is that the space for the former solution is very small, and the search agent needs to constantly adjust the adaptive step size. Therefore, the fix-dimension multimodal benchmark functions can meet the needs of solving different types of problems and can fully evaluate the performance of an optimization algorithm. The formulas of these are shown in Table S3.

### 4.2. Comparison of Test Results with Other Algorithms

We compared the NOL-HHO algorithm with the HHO [34], GA [34], PSO [45], BBO [45], FPA [46], GWO [47], BAT [48], FA [49], MFO [50], and DE [45] optimization algorithms for the mean (AVG) and standard deviation (STD) results of 23 test functions to demonstrate the effectiveness of our improvement. In addition, in order to conduct experiments fairly, all tests were performed under the same conditions, using 30 search agents uniformly in 500 iterations. At the same time, each benchmark function was executed independently 30 times to reduce the randomness of the results, and the specific results are shown in Table 1 and Table 2. To further compare the performance between these algorithms, we studied the effect of dimensions on the quality of the solution and the efficiency of the optimization when the dimension changed. In addition to a 30-dimensional test, the NOL-HHO algorithm and the HHO original algorithm were compared at the 100-, 500-, and 1000-dimensional means and standard deviations, as shown in Table 3. The evaluation criterion was that the closer the mean was to the global optimal value the better and the closer the standard deviation was to 0 the better. The results of all comparison algorithms were taken from the original paper of the HHO algorithm and the best results from all of the above tests are marked in bold.

As can be seen from Table 1, for the unimodal benchmark functions (*F*_1_–*F*_7_), the mean or standard deviation test results of the NOL-HHO algorithm were significantly better than for other algorithms, and functions *F*_1_–*F*_4_ reached a global optimal value in Table S1, indicating that the improved algorithm had a stronger benchmark exploitation capability. In terms of multi-dimensional function testing (*F*_8_–*F*_13_), NOL-HHO also showed superior performance as compared with HHO and other algorithms. The mean and standard deviation of *F*_9_–*F*_11_ reached global optimal values, as shown in Table S2. The mean of the remaining functions of NOL-HHO were closer to the optimal value, and the standard deviation was more stable as compared with the other algorithms. This shows that it had a strong exploration capability. For fix-dimension multimodal benchmark functions (*F*_14_–*F*_23_), it can be seen from Table 2 that the mean was better, except for the function *F*_20_ and the global optimality reached 90%. Functional tests of this different type of problem illustrate the superiority of the overall performance of the NOL-HHO algorithm.

**Figure 5:**
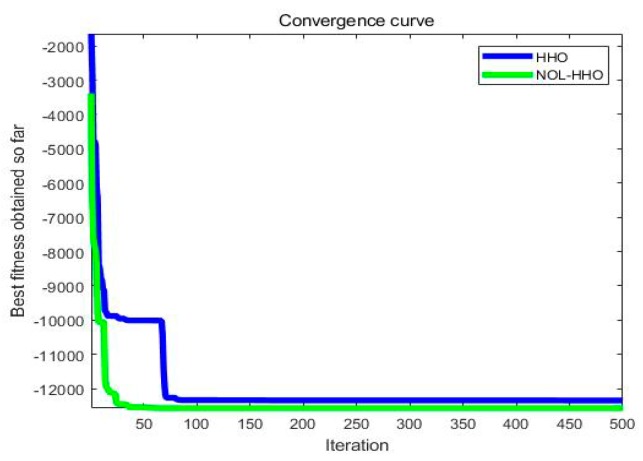
Unimodal test function **F_8_**.

**Figure 6:**
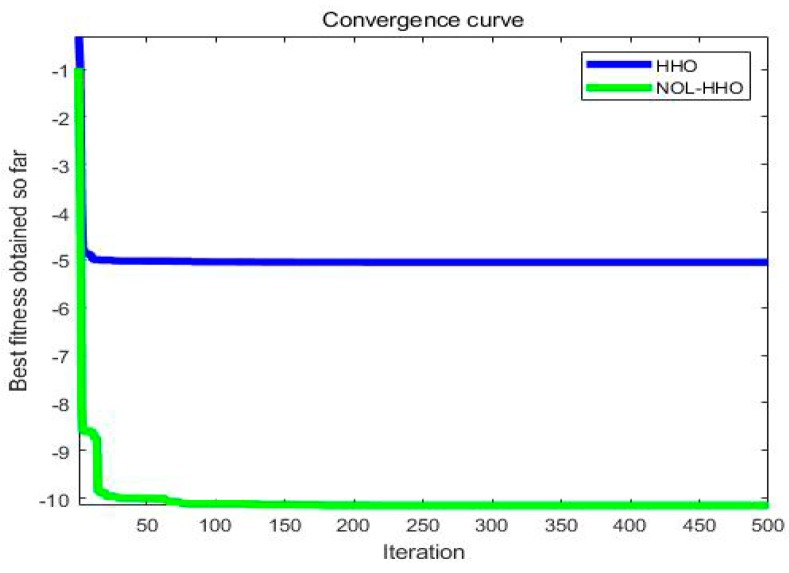
Multimodal test function **F_21_**.

In order to compare the effects before and after the improved HHO algorithm, we drew a fitness curve diagram of the unimodal functions and the multimodal functions, as shown in Figure 5 and Figure 6, using test functions *F*_8_ and *F*_21_. In the unimodal function *F*_8_, although the original algorithm also achieved a global optimal solution, it fell into a local optimum during the search process, while NOL-HHO did not, and the convergence speed was faster for NOL-HHO. The HHO algorithm in the multimodal function *F*_21_ converged on the local area and easily reached a global optimal value after our refinements. This shows that NOL-HHO can maintain a good balance between the exploratory and exploitative nature of dealing with different dimensional problems, and it was better at jumping out of local optima by enriching the population.

In the following, we also tested the mean and standard deviation of different dimensions for the NOL-HHO and HHO algorithms, and the results are shown in Table 3. In the 100-, 500-, and 1000-dimensional tests, NOL-HHO performed 100% better than the original algorithm. This shows that even in high-dimensional situations, NOL-HHO is quite competitive in finding the best solution, and its performance remains consistently superior when realizing cases with many variables.

### 4.3. Wilcoxon Rank-Sum Test

Due to the randomness of the algorithm, the algorithm still required assessment based on additional statistical tests. The results of the mean and standard deviation only show the overall performance of an algorithm, while statistical tests consider whether the results are statistically significant. Therefore, we used the non-parametric Wilcoxon rank-sum test [51] at a 5% significance level [52] to investigate the significant difference between our algorithm results. When P > 0.05 showed that it has significant differences and statistical advantage. Table 4 gives the specific results, and results in bold are optimum values.

We then compared NOL-HHO with the results of the original DA algorithm [53] proposed previously. Thanks to the powerful exploration and jumping-out capabilities of the former, the P-value results were impressive, and its performance was significantly better than DA, PSO, or GA. NOL-HHO had an easier time find promising areas in the search space, again demonstrating that our algorithm outperformed other algorithms as a whole.

## 5. Bound DNA Storage Constraint Coding

In order to test the effect of NOL-HHO on practical problems, we applied it to an optimal constraint coding set in the construction of a DNA storage medium. At the same time, we compared this with the results from last year’s Limbachiya et al. [26], which used an altruistic algorithm. The lower bound of the constrained coding set of 4 ≤ *n* ≤ 10, 3 ≤ *d* ≤ n is shown in Table 5 and the specific meaning of black bold was that the constructed set was better than that of Limbachiya et al. In Table 5, the superscript small a represents the result of Limbachiya, while the superscript R represents our result. A*^GC,NL^ (n, d, w)* represents the set of DNA sequences that satisfies the No-runlength constraint, GC-content constraint, and has a large Hamming distance, where *n* was the length of the sequence, *d* represented the size of the Hamming distance, and *w* referred to the GC-content, typically *n*/2.

We found that except for *n* = 8, 9, or 10 and *d* = 2, the result was not as good as the aforementioned article, while the rest of the results were much better than this article. For example, when *n* = 10 and *d* = 7, the size of our built set was close to three times that of Limbachiya et al. When *n* = 8 and *d* = 5, the result was 25% higher than the previous work. For some of the same results, since global optimization had been achieved, there was no room for further improvement. The combination of a nonlinear convergence factor with powerful exploration capabilities and a jump-out local “ROL” strategy greatly increased the number of DNA constraint code sets, creating the conditions and stable environment for storing large files in DNA.

From the perspective of the coding rate, this was an important factor for measuring the transmission rate in DNA storage. The improvement of the lower bound directly leads to an increase in the coding rate. The code rate is defined as *R = log*_4_ *M/n* [54], where *n* is the length of the DNA code and m is the number of DNA code sets. For example, in the literature [26], for *n* = 9 and *d* = 4, *R = log*_4_ 199/9 ≈ 0.42. Using our method, when *n* = 8 and *d* = 4, it also reached 0.42. In the case of the same code rate, short sequences can achieve the same storage performance as long sequences. A comparison with long DNA sequences, shows that short sequences are easier to synthesize, less expensive, and more stable, which means that the betterment of the lower bound is of great significance for further enhancing the storage performance.

## 6. Conclusions

In this paper, we use an improved HHO algorithm to construct a set of constrained codes in DNA storage. We combined a nonlinear convergence strategy with a random opposition-based learning strategy using the original HHO algorithm, which we termed NOL-HHO. We compared this algorithm with HHO, GA, PSO, GWO, and other algorithms based on 23 test functions, and found that NOL-HHO outperformed other optimizers, demonstrating the effectiveness of our improvements. At the same time, the NOL-HHO was compared with the newly proposed optimizer DA and the classical algorithms, GA and PSO, using a Wilcoxon rank-sum test of 19 test functions. The statistical result P-values confirmed that there was a significant gap between NOL-HHO and the population of other optimizers in almost all cases, indicating that it was more competitive. As a new storage medium, Dong et al. described the work related to DNA storage in detail in the paper [55], which shows that DNA storage has strong development prospects for many practical applications. As a new storage medium, DNA storage has strong development prospects for many practical applications. A constrained coding sequence can guarantee good robustness between sequences and prevent decoding errors. At the same time, an optimally constrained code sequence set needs to be constructed to make it possible to store large files in DNA, and to increase the storage capacity and coding rate. We compared the sequence sets constructed by NOL-HHO with those constructed in previous works and demonstrated that NOL-HHO generates a sequence set with a significantly better lower bound. This should promote further development of DNA storage, using our successful improvement of this algorithm. In addition, the constructed reliable DNA coding set can also be applied to other fields of DNA, such as DNA neural network computing model [56], DNA coding image encryption [57], DNA parallel computing model [58,59], Using the DNA storage architecture to make embedded storage materials [60], etc., which has a wide range of application value.

In the future, we plan to further refine the NOL-HHO algorithm. As far as the results of NOL-HHO are concerned, functions such as *F*_7_ and *F*_20_ were still locally optimal, and there was still room for enhancement. In terms of storage, sequencing remains a major challenge for DNA storage. Lopez et al. [61] successfully proposed and demonstrated a decoding method combining DNA assembly with random access, which greatly improved the throughput of nanopore sequencing, promoted the rapid development of DNA storage, and laid a foundation for our next research work. On the one hand, we will continue to raise the lower bound of the restricted code set and strive to store more information than the lower bounds of previous work. On the other hand, we will work on solving the problems in sequencing. 

## Figures and Tables

**Table 1:** Result of benchmark functions (F_1_–F_13_), with 30 dimensions.

ID	Metric	NOL-HHO	HHO	GA	PSO	BBO	FPA	GWO	BAT	FA	MFO	DE
F_1_	AVG	**0.00 × 10^+00^**	3.95 × 10^−97^	1.03 × 10^+03^	1.83 × 10^+04^	7.59 × 10^+01^	2.01 × 10^+03^	1.18 × 10^−27^	6.59 × 10^+04^	7.11 × 10^−03^	1.01 × 10^+03^	1.33 × 10^−03^
	STD	0.00 × 10^+00^	1.72 × 10^−96^	5.79 × 10^+02^	3.01 × 10^+03^	2.75 × 10^+01^	5.60 × 10^+02^	1.47 × 10^−27^	7.51 × 10^+03^	3.21 × 10^−03^	3.05 × 10^+03^	5.92 × 10^−04^
F_2_	AVG	**0.00 × 10^+00^**	1.56 × 10^−51^	2.47 × 10^+01^	3.58 × 10^+02^	1.36 × 10^−03^	3.22 × 10^+01^	9.71 × 10^−17^	2.71 × 10^+08^	4.34 × 10^−01^	3.19 × 10^+01^	6.83 × 10^−03^
	STD	0.00 × 10^+00^	6.98 × 10^−51^	5.68 × 10^+00^	1.35 × 10^+03^	7.45 × 10^−03^	5.55 × 10^+00^	5.60 × 10^−17^	1.30 × 10^+09^	1.84 × 10^−01^	2.06 × 10^+01^	2.06 × 10^−03^
F_3_	AVG	**0.00 × 10^+00^**	1.92 × 10^−63^	2.65 × 10^+04^	4.05 × 10^+04^	1.21 × 10^+04^	1.41 × 10^+03^	5.12 × 10^−05^	1.38 × 10^+05^	1.66 × 10^+03^	2.43 × 10^+04^	3.97 × 10^+04^
	STD	0.00 × 10^+00^	1.05 × 10^−62^	3.44 × 10^+03^	8.21 × 10^+03^	2.69 × 10^+03^	5.59 × 10^+02^	2.03 × 10^−04^	4.72 × 10^+04^	6.72 × 10^+02^	1.41 × 10^+04^	5.37 × 10^+03^
F_4_	AVG	**0.00 × 10^+00^**	1.02 × 10^−47^	5.17 × 10^+01^	4.39 × 10^+01^	3.02 × 10^+01^	2.38 × 10^+01^	1.24 × 10^−06^	8.51 × 10^+01^	1.11 × 10^−01^	7.00 × 10^+01^	1.15 × 10^+01^
	STD	0.00 × 10^+00^	5.01 × 10^−47^	1.05 × 10^+01^	3.64 × 10^+00^	4.39 × 10^+00^	2.77 × 10^+00^	1.94 × 10^−06^	2.95 × 10^+00^	4.75 × 10^−02^	7.06 × 10^+00^	2.37 × 10^+00^
F_5_	AVG	**1.90 × 10^−03^**	1.32 × 10^−02^	1.95 × 10^+04^	1.96 × 10^+07^	1.82 × 10^+03^	3.17 × 10^+05^	2.70 × 10^+01^	2.10 × 10^+08^	7.97 × 10^+01^	7.35 × 10^+03^	1.06 × 10^+02^
	STD	4.25 × 10^−03^	1.87 × 10^−02^	1.31 × 10^+04^	6.25 × 10^+06^	9.40 × 10^+02^	1.75 × 10^+05^	7.78 × 10^−01^	4.17 × 10^+07^	7.39 × 10^+01^	2.26 × 10^+04^	1.01 × 10^+02^
F_6_	AVG	**6.73 × 10^−09^**	1.15 × 10^−04^	9.01 × 10^+02^	1.87 × 10^+04^	6.71 × 10^+01^	1.70 × 10^+03^	8.44 × 10^−01^	6.69 × 10^+04^	6.94 × 10^−03^	2.68 × 10^+03^	1.44 × 10^−03^
	STD	2.95 × 10^−05^	1.56 × 10^−04^	2.84 × 10^+02^	2.92 × 10^+03^	2.20 × 10^+01^	3.13 × 10^+02^	3.18 × 10^−01^	5.87 × 10^+03^	3.61 × 10^−03^	5.84 × 10^+03^	5.38 × 10^−04^
F_7_	AVG	**7.91 × 10^−05^**	1.40 × 10^−04^	1.91 × 10^−01^	1.07 × 10^+01^	2.91 × 10^−03^	3.41 × 10^−01^	1.70 × 10^−03^	4.57 × 10^+01^	6.62 × 10^−02^	4.50 × 10^+00^	5.24 × 10^−02^
	STD	4.02 × 10^−05^	1.07 × 10^−04^	1.50 × 10^−01^	3.05 × 10^+00^	1.83 × 10^−03^	1.10 × 10^−01^	1.06 × 10^−03^	7.82 × 10^+00^	4.23 × 10^−02^	9.21 × 10^+00^	1.37 × 10^−02^
F_8_	AVG	**−1.26 × 10^+04^**	−1.25 × 10^+04^	**−1.26 × 10^+04^**	−3.86× 10^+03^	−1.24 × 10^+04^	−6.45 × 10^+03^	−5.97 × 10^+03^	−2.33 × 10^+03^	−5.85 × 10^+03^	−8.48 × 10^+03^	−6.82× 10^+03^
	STD	2.17 × 10^−01^	1.47 × 10^+02^	4.51 × 10^+00^	2.49 × 10^+02^	3.50 × 10^+01^	3.03 × 10^+02^	7.10 × 10^+02^	2.96 × 10^+02^	1.16 × 10^+03^	7.98 × 10^+02^	3.94 × 10^+02^
F_9_	AVG	**0.00 × 10^+00^**	**0.00 × 10^+00^**	9.04 × 10^+00^	2.87 × 10^+02^	**0.00 × 10^+00^**	1.82 × 10^+02^	2.19 × 10^+00^	1.92 × 10^+02^	3.82 × 10^+01^	1.59 × 10^+02^	1.58 × 10^+02^
	STD	0.00 × 10^+00^	0.00 × 10^+00^	4.58 × 10^+00^	1.95 × 10^+01^	0.00 × 10^+00^	1.24 × 10^+01^	3.69 × 10^+00^	3.56 × 10^+01^	1.12 × 10^+01^	3.21 × 10^+01^	1.17 × 10^+01^
F_10_	AVG	**8.88 × 10^−16^**	**8.88 × 10^−16^**	1.36 × 10^+01^	1.75 × 10^+01^	2.13 × 10^+00^	7.14 × 10^+00^	1.03 × 10^−13^	1.92 × 10^+01^	4.58 × 10^−02^	1.74 × 10^+01^	1.21 × 10^−02^
	STD	0.00 × 10^+00^	4.01 × 10^−31^	1.51 × 10^+00^	3.67 × 10^−01^	3.53 × 10^−01^	1.08 × 10^+00^	1.70 × 10^−14^	2.43 × 10^−01^	1.20 × 10^−02^	4.95 × 10^+00^	3.30 × 10^−03^
F_11_	AVG	**0.00 × 10^+00^**	**0.00 × 10^+00^**	1.01 × 10^+01^	1.70 × 10^+02^	1.46 × 10^+00^	1.73 × 10^+01^	4.76 × 10^−03^	6.01 × 10^+02^	4.23 × 10^−03^	3.10 × 10^+01^	3.52 × 10^−02^
	STD	0.00 × 10^+00^	0.00 × 10^+00^	2.43 × 10^+00^	3.17 × 10^+01^	1.69 × 10^−01^	3.63 × 10^+00^	8.57 × 10^−03^	5.50 × 10^+01^	1.29 × 10^−03^	5.94 × 10^+01^	7.20 × 10^−02^
F_12_	AVG	**3.17 × 10^−07^**	2.08 × 10^−06^	4.77 × 10^+00^	1.51 × 10^+07^	6.68 × 10^−01^	3.05 × 10^+02^	4.83 × 10^−02^	4.71 × 10^+08^	3.13 × 10^−04^	2.46 × 10^+02^	2.25 × 10^−03^
	STD	5.24 × 10^-07^	1.19 × 10^−05^	1.56 × 10^+00^	9.88 × 10^+06^	2.62 × 10^−01^	1.04 × 10^+03^	2.12 × 10^−02^	1.54 × 10^+08^	1.76 × 10^−04^	1.21 × 10^+03^	1.70 × 10^−03^
F_13_	AVG	**2.27 × 10^−06^**	1.57 × 10^−04^	1.52 × 10^+01^	5.73 × 10^+07^	1.82 × 10^+00^	9.59 × 10^+04^	5.96 × 10^−01^	9.40 × 10^+08^	2.08 × 10^−03^	2.73 × 10^+07^	9.12 × 10^−03^
	STD	1.42 × 10^−05^	2.15 × 10^−04^	4.52 × 10^+00^	2.68 × 10^+07^	3.41 × 10^−01^	1.46 × 10^+05^	2.23 × 10^−01^	1.67 × 10^+08^	9.62 × 10^−04^	1.04 × 10^+08^	1.16 × 10^−02^

**Table 2:** Results of benchmark functions (F_14_–F_23_), with 30 dimensions.

ID	Metric	NOL-HHO	HHO	GA	PSO	BBO	FPA	GWO	BAT	FA	MFO	DE
**F_14_**	AVG	**9.98 × 10^−01^**	**9.98 × 10^−01^**	**9.98 × 10^−01^**	1.39 × 10^+00^	**9.98 × 10^−01^**	**9.98 × 10^−01^**	4.17 × 10^+00^	1.27 × 10^+01^	3.51 × 10^+00^	2.74 × 10^+00^	1.23 × 10^+00^
	STD	4.77 × 10^−01^	9.23 × 10^−01^	4.52 × 10^−16^	4.60 × 10^−01^	4.52 × 10^−16^	2.00 × 10^−04^	3.61 × 10^+00^	6.96 × 10^+00^	2.16 × 10^+00^	1.82 × 10^+00^	9.23 × 10^−01^
**F_15_**	AVG	**3.08 × 10^−04^**	3.10 × 10^−04^	3.33 × 10^−02^	1.61 × 10^−03^	1.66 × 10^−02^	6.88 × 10^−04^	6.24 × 10^−03^	3.00 × 10^−02^	1.01 × 10^−03^	2.35 × 10^−03^	5.63 × 10^−04^
	STD	4.23 × 10^−05^	1.97 × 10^−04^	2.70 × 10^−02^	4.60 × 10^−04^	8.60 × 10^−03^	1.55 × 10^−04^	1.25 × 10^−02^	3.33 × 10^−02^	4.01 × 10^−04^	4.92 × 10^−03^	2.81 × 10^−04^
**F_16_**	AVG	**−1.03 × 10^+00^**	**−1.03 × 10^+00^**	−3.78 × 10^−01^	**−1.03 × 10^+00^**	−8.30 × 10^−01^	**−1.03 × 10^+00^**	**−1.03 × 10^+00^**	−6.87 × 10^−01^	**−1.03 × 10^+00^**	**−1.03 × 10^+00^**	**−1.03 × 10^+00^**
	STD	1.42 × 10^−08^	6.78 × 10^−16^	3.42 × 10^−01^	2.95 × 10^−03^	3.16 × 10^−01^	6.78 × 10^−16^	6.78 × 10^−16^	8.18 × 10^−01^	6.78 × 10^−16^	6.78 × 10^−16^	6.78 × 10^−16^
**F_17_**	AVG	**3.98 × 10^−01^**	**3.98 × 10^−01^**	5.24 × 10^−01^	4.00 × 10^−01^	5.49 × 10^−01^	**3.98 × 10^−01^**	**3.98 × 10^−01^**	**3.98 × 10^−01^**	**3.98 × 10^−01^**	**3.98 × 10^−01^**	**3.98 × 10^−01^**
	STD	4.92 × 10^−06^	2.54 × 10^−06^	6.06 × 10^−02^	1.39 × 10^−03^	6.05 × 10^−02^	1.69 × 10^−16^	1.69 × 10^−16^	1.58 × 10^−03^	1.69 × 10^−16^	1.69 × 10^−16^	1.69 × 10^−16^
**F_18_**	AVG	**3.00 × 10^+00^**	**3.00 × 10^+00^**	**3.00 × 10^+00^**	3.10 × 10^+00^	**3.00 × 10^+00^**	**3.00 × 10^+00^**	**3.00 × 10^+00^**	1.47 × 10^+01^	**3.00 × 10^+00^**	**3.00 × 10^+00^**	**3.00 × 10^+00^**
	STD	7.69 × 10^−07^	0.00 × 10^+00^	0.00 × 10^+00^	7.60 × 10_−02_	0.00 × 10^+00^	0.00 × 10^+00^	4.07 × 10^−05^	2.21 × 10^+01^	0.00 × 10^+00^	0.00 × 10^+00^	0.00 × 10^+00^
**F_19_**	AVG	**−3.86 × 10^+00^**	**−3.86 × 10^+00^**	−3.42 × 10^+00^	**−3.86 × 10^+00^**	−3.78 × 10^+00^	**−3.86 × 10^+00^**	**−3.86 × 10^+00^**	−3.84 × 10^+00^	**−3.86 × 10^+00^**	**−3.86 × 10^+00^**	**−3.86 × 10^+00^**
	STD	1.43 × 10^−03^	2.44 × 10^−03^	3.03 × 10^−01^	1.24 × 10^−03^	1.26 × 10^−01^	3.16 × 10^−15^	3.14 × 10^−03^	1.41 × 10^−01^	3.16 × 10^−15^	1.44 × 10^−03^	3.16 × 10^−15^
**F_20_**	AVG	−3.260	**−3.322**	−1.61351	−3.11088	−2.70774	−3.2951	−3.25866	−3.2546	−3.28105	−3.23509	−3.27048
	STD	0.115550	0.137406	0.46049	0.029126	0.357832	0.019514	0.064305	0.058943	0.063635	0.064223	0.058919
**F_21_**	AVG	**−10.1532**	−10.1451	−6.66177	−4.14764	−8.31508	−5.21514	−8.64121	−4.2661	−7.67362	−6.8859	−9.64796
	STD	0.001000	0.885673	3.732521	0.919578	2.883867	0.008154	2.563356	2.554009	3.50697	3.18186	1.51572
**F_22_**	AVG	**−10.4028**	−10.4015	−5.58399	−6.01045	−9.38408	−5.34373	−10.4014	−5.60638	−9.63827	−8.26492	−9.74807
	STD	0.000850	1.352375	2.605837	1.962628	2.597238	0.053685	0.000678	3.022612	2.293901	3.076809	1.987703
**F_23_**	AVG	**−10.5364**	**−10.5364**	−4.69882	−4.72192	−6.2351	−5.29437	−10.0836	−3.97284	−9.75489	−7.65923	**−10.5364**
	STD	0.000940	0.927655	3.256702	1.742618	3.78462	0.356377	1.721889	3.008279	2.345487	3.576927	8.88 × 10^−15^

**Table 3:** NOL-HHO results for different dimensions using functions F_1_–F_13_.

ID	Metric	NOL-HHO (100dim)	HHO (100 dim)	NOL-HHO (500 dim)	HHO (500 dim)	NOL-HHO (1000 dim)	HHO (1000 dim)
**F_1_**	AVG	**0.00 × 10^+00^**	1.91 × 10^−94^	**0.00 × 10^+00^**	1.46 × 10^−92^	**0.00 × 10^+00^**	1.06 × 10^−94^
	STD	0.00 × 10^+00^	8.66 × 10^−94^	0.00 × 10^+00^	8.01 × 10^−92^	0.00 × 10^+00^	4.97 × 10^−94^
**F_2_**	AVG	**0.00 × 10^+00^**	9.98 × 10^−52^	**0.00 × 10^+00^**	7.87 × 10^−49^	**0.00 × 10^+00^**	2.52 × 10^−50^
	STD	0.00 × 10^+00^	2.66 × 10^−51^	0.00 × 10^+00^	3.11 × 10^−48^	0.00 × 10^+00^	5.02 × 10^−50^
**F_3_**	AVG	**0.00 × 10^+00^**	1.84 × 10^−59^	**0.00 × 10^+00^**	6.54 × 10^−37^	**0.00 × 10^+00^**	1.79 × 10^−17^
	STD	0.00 × 10^+00^	1.01 × 10^−58^	0.00 × 10^+00^	3.58 × 10^−36^	0.00 × 10^+00^	9.81 × 10^−17^
**F_4_**	AVG	**0.00 × 10^+00^**	8.76 × 10^−47^	**0.00 × 10^+00^**	1.29 × 10^−47^	**0.00 × 10^+00^**	1.43 × 10^−46^
	STD	0.00 × 10^+00^	4.79 × 10^−46^	0.00 × 10^+00^	4.11 × 10^−47^	0.00 × 10^+00^	7.74 × 10^−46^
**F_5_**	AVG	**1.22 × 10^−04^**	2.36 × 10^−02^	**6.66 × 10^−02^**	3.10 × 10^−01^	**2.29 × 10^−02^**	5.73 × 10^−01^
	STD	5.95 × 10^−03^	2.99 × 10^−02^	4.79 × 10^−02^	3.73 × 10^−01^	1.01 × 10^−01^	1.40 × 10^+00^
**F_6_**	AVG	**7.71 × 10^−06^**	5.12 × 10^−04^	**6.85 × 10^−06^**	2.94 × 10^−03^	**1.81 × 10^−04^**	3.61 × 10^−03^
	STD	6.62 × 10^−05^	6.77 × 10^−04^	8.04 × 10^−04^	3.98 × 10^−01^	1.02 × 10^−03^	5.38 × 10^−03^
**F_7_**	AVG	**6.59 × 10^−05^**	1.85 × 10^−04^	**2.24 × 10^−05^**	2.51 × 10^−04^	**3.81 × 10^−06^**	1.41 × 10^−04^
	**STD**	**3.63 × 10^−05^**	**4.06 × 10^−04^**	**2.87 × 10^−05^**	**2.43 × 10^−04^**	**5.79 × 10^−05^**	**1.63 × 10^−04^**
**F_8_**	AVG	**−4.19 × 10^+04^**	**−4.19 × 10^+04^**	**−2.09 × 10^+05^**	**−2.09 × 10^+05^**	**−4.19 × 10^+05^**	**−4.19 × 10^+05^**
	STD	1.35 × 10^+00^	2.82 × 10^+00^	8.13 × 10^+00^	2.84 × 10^+01^	1.01 × 10^+01^	1.03 × 10^+02^
**F_9_**	AVG	**0.00 × 10^+00^**	**0.00 × 10^+00^**	**0.00 × 10^+00^**	**0.00 × 10^+00^**	**0.00 × 10^+00^**	**0.00 × 10^+00^**
	STD	0.00 × 10^+00^	0.00 × 10^+00^	0.00 × 10^+00^	0.00 × 10^+00^	0.00 × 10^+00^	0.00 × 10^+00^
**F_10_**	AVG	**8.88 × 10^−16^**	**8.88 × 10^−16^**	**8.88 × 10^−16^**	**8.88 × 10^−16^**	**8.88 × 10^−16^**	**8.88 × 10^−16^**
	STD	0.00 × 10^+00^	4.01 × 10^−31^	0.00 × 10^+00^	4.01 × 10^−31^	0.00 × 10^+00^	4.01 × 10^−31^
**F_11_**	AVG	**0.00 × 10^+00^**	**0.00 × 10^+00^**	**0.00 × 10^+00^**	**0.00 × 10^+00^**	**0.00 × 10^+00^**	**0.00 × 10^+00^**
	STD	0.00 × 10^+00^	0.00 × 10^+00^	0.00 × 10^+00^	0.00 × 10^+00^	0.00 × 10^+00^	0.00 × 10^+00^
**F_12_**	AVG	**2.62 × 10^−09^**	4.23 × 10^−06^	**1.17 × 10^−10^**	1.41 × 10^−06^	**1.18 × 10^−08^**	1.02 × 10^−06^
	STD	4.18 × 10^−07^	5.25× 10^−06^	1.02 × 10^−07^	1.48 × 10^−06^	1.94 × 10^−07^	1.16 × 10^−06^
**F_13_**	AVG	**6.46 × 10^−06^**	9.13 × 10^−05^	**6.47 × 10^−05^**	3.44 × 10^−04^	**2.63 × 10^−04^**	8.41 × 10^−04^
	STD	1.45 × 10^−05^	1.26 × 10^−04^	1.09 × 10^−04^	4.75 × 10^−04^	1.92 × 10^−04^	1.18 × 10^−03^

**Table 4:** P-values of the Wilcoxon rank-sum test over all runs.

F	NOL-HHO	DA	GA	PSO
**F_1_**	**0.086927**	N/A	0.000183	0.045155
**F_2_**	N/A	N/A	0.000183	**0.121225**
**F_3_**	**0.198360**	N/A	0.000183	0.003611
**F_4_**	N/A	N/A	0.000183	**0.307489**
**F_5_**	**0.540770**	N/A	0.000183	0.10411
**F_6_**	**0.470240**	0.344704	0.000183	N/A
**F_7_**	0.000393	**0.021134**	0.000183	N/A
**F_8_**	**0.156580**	0.000183	0.000183	N/A
**F_9_**	0.091461	**0.364166**	0.002202	N/A
**F_10_**	0.076666	N/A	0.000183	**0.472676**
**F_11_**	**0.060926**	0.001008	0.000183	N/A
**F_12_**	**0.26758**	0.140465	0.000183	N/A
**F_13_**	0.111660	N/A	0.000183	**0.79126**

**Table 5:** Lower bounds for A*^GC,NL^ (n, d, w)*. (*K* represents our work, *a* represents Limbachiya’s work).

n\d	2	3	4	5	6	7	8	9	10
4	32 *^a^*	11 *^a^*							
	32 ***^K^***	**12 *^K^***	**4 *^K^***						
5	68 *^a^*	17 *^a^*	7 *^a^*	2 *^a^*					
	68 ***^K^***	**20 *^K^***	**8 *^K^***	**3 *^K^***					
6	216 *^a^*	44 *^a^*	16 *^a^*	6 *^a^*	4 *^a^*				
	216 ***^K^***	**55 *^K^***	**23 *^K^***	**8 *^K^***	4 ***^K^***				
7	528 *^a^*	110 *^a^*	36 *^a^*	11 *^a^*	4 *^a^*	2 *^a^*			
	528 ***^K^***	**121 *^K^***	**42 *^K^***	**14 *^K^***	**7 *^K^***	**3 *^K^***			
8	1704 *^a^*	289 *^a^*	86 *^a^*	29 *^a^*	9 *^a^*	4 *^a^*	4 *^a^*		
	1694 ***^K^***	**339 *^K^***	**108 *^K^***	**35 *^K^***	**13 *^K^***	**5 *^K^***	4 ***^K^***		
9	4336 *^a^*	662 ^*a*^	199 ^*a*^	59 *^a^*	15 *^a^*	8 *^a^*	4 *^a^*	4 *^a^*	
	4310 ***^K^***	**705 *^K^***	**216 *^K^***	**69 *^K^***	**22 *^K^***	**11 *^K^***	4 ***^K^***	3 ***^K^***	
10	13,688 *^a^*	1810 *^a^*	525 *^a^*	141 *^a^*	43 *^a^*	7 *^a^*	5 *^a^*	4 *^a^*	4 *^a^*
	13,410 ***^K^***	1796 ***^K^***	**546 *^K^***	**148 *^K^***	**51 *^K^***	**20 *^K^***	**9 *^K^***	4 ***^K^***	4 ***^K^***

## Supplementary Materials

The following are available online at https://www.mdpi.com/1422-0067/21/6/2191/s1.

## References

[1] Wang Y., Noor-A-Rahim M.D., Gunawan E., Guan Y., Poh C.L. (2019). Construction of bio-constrained code for DNA data storage. IEEE Commun. Lett..

[2] Li D., Wang Y., Noor-A-Rahim M.D., Guan Y., Shi Z., Gunawan E. (2019). Optimized code design for constrained DNA data storage with asymmetric errors. IEEE Access.

[3] Ping Z., Ma D., Huang X., Chen S., Liu L., Guo F., Zhu S.J., Shen Y. (2019). Carbon-based archiving: Current progress and future prospects of DNA-based data storage. GigaScience.

[4] Church G.M., Gao Y., Kosuri S. (2012). Next-generation digital information storage in DNA. Science.

[5] Zhang S., Huang B., Song X., Zhang T., Wang H., Liu Y. (2019). A high storage density strategy for digital information based on synthetic DNA. 3 Biotech.

[6] Goldman N., Bertone P., Chen S., Dessimoz C., Leproust E.M., Sipos B. (2013). Towards practical, high-capacity, low-maintenance information storage in synthesized DNA. Nature.

[7] Erlich Y., Zielinski D. (2017). DNA Fountain enables a robust and efficient storage architecture. Science.

[8] Palluk S., Arlow D.H., Rond T.D., Barthel S., Kang J.S., Bector R. (2018). De novo DNA synthesis using polymerase-nucleotide conjugates. Nat. Biotechnol..

[9] Shendure J., Balasubramanian S., Church G.M., Gilbert W., Rogers J., Schloss J.A. (2017). DNA sequencing at 40: Past, present and future. Nature.

[10] Baum E.B. (1995). Building an associative memory vastly larger than the brain. Science.

[11] Clelland C.T., Risca V., Bancroft C. (1999). Hiding messages in DNA microdots. Nature.

[12] Bancroft C., Bowler T., Bloom B., Clelland C.T. (2001). Long-Term Storage of Information in DNA. Science.

[13] Kashiwamura S., Yamamoto M., Kameda A., Shiba T., Ohuchi A. (2005). Potential for enlarging DNA memory: The validity of experimental operations of scaled-up nested primer molecular memory. BioSystems.

[14] Ailenberg M., Rotstein O. (2009). An improved Huffman coding method for archiving text, images, and music characters in DNA. BioTechniques.

[15] Yazdi S.M.H.T., Yuan Y., Ma J., Zhao H., Milenkovic O. (2015). A rewritable, random-access DNA-based storage system. Sci. Rep..

[16] Bornholt J., Lopez R., Carmean D.M., Ceze L., Seelig G., Strauss K. (2016). A DNA-based archival storage system. Archit. Support Program. Lang. Oper. Syst..

[17] Blawat M., Gaedke K., Hütter I., Chen X., Turczyk B., Inverso S. (2016). Forward error correction for DNA data storage. Int. Conf. Concept. Struct..

[18] Yazdi S.M.H.T., Gabrys R., Milenkovic O. (2017). Portable and Error-Free DNA-Based Data Storage. Sci. Rep..

[19] Gabrys R., Kiah H.M., Milenkovic O. (2017). Asymmetric Lee distance codes for DNA-based storage. IEEE Trans. Inf. Theory.

[20] Immink K.A.S., Cai K. (2018). Design of capacity-approaching constrained codes for DNA-based storage systems. IEEE Commun. Lett..

[21] Organick L., Ang S.D., Chen Y.J., Lopez R., Yekhanin S., Makarychev K. (2018). Random access in large-scale DNA data storage. Nat. Biotechnol..

[22] Yazdi S.M.H.T., Kiah H.M., Gabrys R., Milenkovic O. (2018). Mutually uncorrelated primers for DNA-based data storage. IEEE Trans. Inf. Theory..

[23] Song W., Cai K., Zhang M., Yuen C. (2018). Codes with run-length and GC-content constraints for DNA-based data storage. IEEE Commun. Lett..

[24] Carmean D., Ceze L., Seelig G., Stewart K., Strauss K., Willsey M. (2018). DNA data storage and hybrid molecular –electronic computing. Proc. IEEE.

[25] Heckel R., Mikutis G., Grass R.N. (2019). A Characterization of the DNA Data storage Channel. Sci. Rep..

[26] Limbachiya D., Gupta M.K., Aggarwal V. (2018). Family of constrained codes for archival DNA data storage. IEEE Commun. Lett..

[27] Takahashi C.N., Nguyen B.H., Strauss K., Ceze L. (2019). Demonstration of end-to-end automation of DNA data storage. Sci. Rep..

[28] Sun J., Wang Q., Diao W., Zhou C., Wang B., Rao L. (2019). Digital information storage on DNA in living organisms. Med Res. Arch..

[29] Ceze L., Nivala J., Strauss K. (2019). Molecular digital data storage using DNA. Nat. Rev. Genet..

[30] Wang Y., Keith M., Leyme A., Bergelson S., Feschenko M. (2019). Monitoring long-term DNA storage via absolute copy number quantification by ddPCR. Anal. Biochem..

[31] Anavy L., Vaknin I., Atar O., Amit R., Yakhini Z. (2019). Data storage in DNA with fewer synthesis cycles using composite DNA letters. Nat. Biotechnol..

[32] Li X., Wang B., Lv H., Yin Q., Zhang Q., Wei X. (2020). Constraining DNA sequences with a triplet-bases unpaired. IEEE Trans. NanoBiosci..

[33] Wang B., Zhang Q., Wei X. (2020). Tabu Variable Neighborhood Search for Designing DNA Barcodes. IEEE Trans. NanoBiosci..

[34] Heidari A.A., Mirjalili S., Faris H., Aljarah I., Mafarja M., Chen H. (2019). Harris hawks optimization: Algorithm and applications. Future Gener. Comput. Syst..

[35] Bui D.T., Moayedi H., Kalantar B., Osouli A., Pradhan B., Nguyen H. (2019). A novel swarm intelligence—Harris hawks optimization for spatial assessment of landslide susceptibility. Sensors.

[36] Jia H., Lang C., Oliva D., Song W., Peng X. (2019). Dynamic Harris Hawks Optimization with Mutation Mechanism for Satellite Image Segmentation. Remote Sens..

[37] Bao X., Jia H., Lang C. (2019). A Novel Hybrid Harris Hawks Optimization for Color Image Multilevel Thresholding Segmentation. IEEE Access.

[38] Teng Z., Lv J., Guo L. (2019). An improved hybrid grey wolf optimization algorithm. Soft Comput..

[39] Tizhoosh H.R. Opposition-Based Learning: A New Scheme for Machine Intelligence. Proceedings of the International Conference on Computational Intelligence for Modelling, Control and Automation and International Conference on Intelligent Agents, Web Technologies and Internet Commerce (CIMCA-IAWTIC’06).

[40] Banerjee A., Herjee V.M., Ghoshal S.P. (2014). An opposition-based harmony search algorithm for engineering optimization problems. Ain Shams Eng. J..

[41] Dong W., Kang L., Zhang W. (2017). Opposition-based particle swarm optimization with adaptive mutation strategy. Soft Comput..

[42] Ibrahim R.A., Elaziz M.A., Oliva D., Cuevas E., Lu S. (2019). An opposition-based social spider optimization for feature selection. Soft Comput..

[43] Digalakis J.G., Margaritis K.G. (2000). On benchmarking functions for genetic algorithms. Int. J. Comput. Math..

[44] Yao X., Liu Y., Lin G. (1999). Evolutionary programming made faster. IEEE Trans. Evol. Comput..

[45] Simon D. (2008). Biogeography-Based Optimization. IEEE Trans. Evol. Comput..

[46] Yang X., Karamanoglu M., He X. (2013). Flower pollination algorithm: A novel approach for multiobjective optimization. Eng. Optim..

[47] Mirjalili S., Mirjalili S.M., Lewis A. (2014). Grey Wolf Optimizer. Adv. Eng. Softw..

[48] Yang X., Gandomi A.H. (2012). Bat algorithm: A novel approach for global engineering optimization. Eng. Comput..

[49] Gandomi A.H., Yang X., Alavi A.H. (2011). Mixed variable structural optimization using Firefly Algorithm. Comput. Struct..

[50] Mirjalili S. (2015). Moth-flame optimization algorithm. Knowl. -Based Syst..

[51] Cao B., Zhao S., Li X., Wang B. (2020). K-means Multi-Verse Optimizer (KMVO) Algorithm to Construct DNA Storage Codes. IEEE Access.

[52] Derrac J., García S., Molina D., Herrera F. (2011). A practical tutorial on the use of nonparametric statistical tests as a methodology for comparing evolutionary and swarm intelligence algorithms. Swarm Evol. Comput..

[53] Mirjalili S. (2016). Dragonfly algorithm: A new meta-heuristic optimization technique for solving single-objective, discrete, and multi-objective problems. Neural Comput. Appl..

[54] Limbachiya D., Dhameliya V., Khakhar M., Gupta M.K. (2015). On optimal family of codes for archival DNA storage. 2015 Seventh International Workshop on Signal Design and Its Applications in Communications (IWSDA).

[55] Dong Y., Sun F., Ping Z., Ouyang Q., Qian L. (2020). DNA storage: Research landscape and future prospects. Natl. Sci. Rev..

[56] Song T., Rodriguez-Paton A., Zheng P., Zeng X. (2018). Spiking neural P systems with colored spikes. IEEE Trans. Cogn. Dev. Syst..

[57] Wang B., Xie Y., Zhou S., Zheng X., Zhou C. (2018). Correcting errors in image encryption based on DNA coding. Molecules.

[58] Song T., Pang S., Hao S., Rodríguez-Patón A., Zheng P. (2019). A parallel image skeletonizing method using spiking neural P systems with weights. Neural Process. Lett..

[59] Song T., Pan L., Wu T., Zheng P., Wong M.L.D., Rodriguez-Paton A. (2019). Spiking neural P systems with learning functions. IEEE Trans. NanoBiosci..

[60] Koch J., Gantenbein S., Masania K., Stark W.J., Erlich Y., Grass R.N. (2020). A DNA-of-things storage architecture to create materials with embedded memory. Nat. Biotechnol..

[61] Lopez R., Chen Y.J., Ang S.D., Yekhanin S., Makarychev K., Racz M.Z., Seelig G., Strauss K., Ceze L. (2019). DNA assembly for nanopore data storage readout. Nat. Commun..

