# Supplementary material for: An Intelligent Optimization Algorithm for Constructing a DNA Storage Code: NOL-HHO
Source: Int J Mol Sci. 2020 Mar 22;21(6):2191. doi: 10.3390/ijms21062191 (PMC7139338; doi:10.3390/ijms21062191)
Supplement: Supplementary file 1 [file ijms-21-02191-s001.pdf]

Table S1. Description of Uni-modal benchmark functions

Function	Dim	Range	f_{min}
$F_1(x)=\sum_{i=1}^{n} X_i^2$	50	[-100,100]	0
$F_2(x)=\sum_{i=1}^{n} \lvert x_i \rvert+\prod_{i=1}^{n} \lvert x_i \rvert$	50	[-10,10]	0
$F_3(x)=\sum_{i=1}^{n}(\sum_{j-1}^{i} x_j)^2$	50	[-100,100]	0
$F_4(x)=\max_i\{\lvert x_i \rvert, 1\le i\le n\}$	50	[-100,100]	0
$F_5(x)=\sum_{i=1}^{n-1}[100(x_{i+1}-x_i^2)^2+(x_i-1)^2]$	50	[-30,30]	0
$F_6(x)=\sum_{i=1}^{n}([x_i+0.5])^2$	50	[-100,100]	0
$F_7(x)=\sum_{i=1}^{n} ix_i^4+random[0,1)$	50	[-1.28,1.28]	0

Table S2. Description of Multi-modal benchmark functions.

Function	Dim	Range	f_{min}
$F_8(x)=\sum_{i=1}^{n}-x_i\sin(\sqrt{\lvert x_i \rvert})$	50	[-500,500]	-418.9829*5
$F_9(x)=\sum_{i=1}^{n}[x_i^2-10\cos(2\pi x_i)+10]$	50	[-5.12,5.12]	0
$F_{10}(x)=-20\exp(-0.2\sqrt{\frac{1}{n}\sum_{i=1}^{n}x_i^2})-\exp\left(\frac{1}{n}\sum_{i=1}^{n}\cos(2\pi x_i)\right)+20+e$	50	[-32,32]	0
$F_{11}(x)=\frac{1}{4000}\sum_{i=1}^{n}x_i^2-\prod_{i=1}^{n}x_i^2\cos\left(\frac{x_i}{\sqrt{i}}\right)+1$	50	[-600,600]	0
$F_{12}(x)=\frac{\pi}{n}\left\{10\sin(\pi y_1)+\sum_{i=1}^{n-1}(y_i-1)^2[1+10\sin^2(\pi y_{i+1})+(y_n-1)^2]\right\}+\sum_{i=1}^{n}u(x_i,10,100,4)$; $y_i=1+\frac{x_i+1}{4}$; $u(x_i,a,k,m)=\begin{cases}k(x_i-a)^m & x_i>a\\ 0 & -a<x_i<a\\ k(-x_i-a)^m & x_i<-a\end{cases}$	50	[-50,50]	0
$F_{13}(x)=0.1\left\{\sin^2(3\pi x_1)+\sum_{i=1}^{n}\begin{matrix}(x_i-1)^2[1+\sin^2(3\pi x_i+1)]+\\(x_n-1)^2[1+\sin^2(2\pi x_n)]\end{matrix}\right\}$	50	[-50,50]	0

Table S3. Description of Fixed-dimension Multi-modal benchmark functions.

Function	Dim	Range	f_{min}
$F_{14}(x)=(\frac{1}{500}+\sum_{j=1}^{25}\frac{1}{j+\sum_{i=1}^{2}(x_i-a_{ij})})^{-1}$	2	[-65,65]	1
$F_{15}(x)=\sum_{i=1}^{11}[a_i-\frac{x_1(b_i^2+b_ix_2)}{b_i^2+b_ix_3+x_4}]^2$	4	[-5,5]	0.00030
$F_{16}(x)=4x_1^2-2.1x_1^4+\frac{1}{3}x_1^6+x_1x_2-4x_2^2+4x_2^4$	2	[-5,5]	-1.0316
$F_{17}(x)=\left(x_2-\frac{5.1}{4\pi^2}x_1^2+\frac{5}{\pi}x_1-6\right)^2+10\left(1-\frac{1}{8\pi}\right)\cos x_1+10$	2	[-5,5]	0.398
$F_{18}(x)=\left[1+(x_1+x_2+1)^2(19-14x_1+3x_1^2-14x_2+6x_1x_2+3x_2^2)\right]$ $\times\left[30+(2x_1-3x_2)^2\times(18-32x_1+12x_1^2+48x_2-36x_1x_2+27x_2^2)\right]$	2	[-2,2]	3
$F_{19}(x)=-\sum_{i=1}^{4}c_i\exp\left(-\sum_{j=1}^{3}a_{ij}\left(x_j-p_{ij}\right)^2\right)$	3	[1,3]	-3.86
$F_{20}(x)=-\sum_{i=1}^{4}c_i\exp\left(-\sum_{j=1}^{6}a_{ij}\left(x_j-p_{ij}\right)^2\right)$	6	[0,1]	-3.32
$F_{21}(x)=-\sum_{i=1}^{5}\left[\left(X-a_i\right)\left(X-a_i\right)^T+c_i\right]^{-1}$	4	[0,10]	-10.1532
$F_{22}(x)=-\sum_{i=1}^{7}\left[\left(X-a_i\right)\left(X-a_i\right)^T+c_i\right]^{-1}$	4	[0,10]	-10.4028
$F_{23}(x)=-\sum_{i=1}^{10}\left[\left(X-a_i\right)\left(X-a_i\right)^T+c_i\right]^{-1}$	4	[0,10]	-10.5363

Table 2. Description of Multi-modal benchmark functions.

Function	Dim	Range	f_{min}
$F_8(x)=\sum_{i=1}^{n}-x_i\sin(\sqrt{\lvert x_i \rvert})$	50	[-500,500]	-418.9829*5
$F_9(x)=\sum_{i=1}^{n}[x_i^2-10\cos(2\pi x_i)+10]$	50	[-5.12,5.12]	0
$F_{10}(x)=-20\exp(-0.2\sqrt{\frac{1}{n}\sum_{i=1}^{n}x_i^2})-\exp\left(\frac{1}{n}\sum_{i=1}^{n}\cos(2\pi x_i)\right)+20+e$	50	[-32,32]	0
$F_{11}(x)=\frac{1}{4000}\sum_{i=1}^{n}x_i^2-\prod_{i=1}^{n}x_i^2\cos\left(\frac{x_i}{\sqrt{i}}\right)+1$	50	[-600,600]	0
$F_{12}(x)=\frac{\pi}{n}\left\{10\sin(\pi y_1)+\sum_{i=1}^{n-1}(y_i-1)^2[1+10\sin^2(\pi y_{i+1})+(y_n-1)^2]\right\}+\sum_{i=1}^{n}u(x_i,10,100,4)$ $y_i=1+\frac{x_i+1}{4}$ $u(x_i,a,k,m)=\begin{cases}k(x_i-a)^m & x_i>a\\ 0 & -a<x_i<a\\ k(-x_i-a)^m & x_i<-a\end{cases}$	50	[-50,50]	0
$F_{13}(x)=0.1\left\{\sin^2(3\pi x_1)+\sum_{i=1}^{n}\begin{matrix}(x_i-1)^2[1+\sin^2(3\pi x_i+1)]+\\(x_n-1)^2[1+\sin^2(2\pi x_n)]\end{matrix}\right\}$	50	[-50,50]	0
